# Imaging of the intrinsic muscles of the hand – Part II: Pathological aspects on high-resolution ultrasound and 3T MRI

**DOI:** 10.1055/a-2735-2780

**Published:** 2026-01-08

**Authors:** Hicham Bouredoucen, Sana Boudabbous, Pierre-Alexandre Poletti, Lokmane Taihi

**Affiliations:** 1 27230 Imaging and Medical Informatics, Geneva University Hospitals, Geneva, Switzerland; 2 70492 Medical Imaging, University Hospital Saint-Luc, Brussels, Belgium

**Keywords:** Intrinsic muscles of the hand, Muscular denervation, Carpal Tunnel Syndrome, Guyon’s Canal Syndrome, Myositis, MRI imaging

## Abstract

**Background:**

There are numerous pathologies of the intrinsic muscles of the hand (IMH). Muscle denervation syndromes of the hand are common in pathologies of the median nerve (MN) and ulnar nerve (UN). They occur following carpal tunnel syndrome, Guyon’s canal syndrome, traumatic, tumoral, inflammatory, or proximal nerve lesions due to upper cervical radiculopathy, or brachial plexus lesions. Myositis of the IMH occurs in various contexts. Traumatic muscle injuries of the hand are rare. Some are characteristic, such as lesions of the third interosseous muscle (IOM) or lesions of the lumbrical muscles (LM) in climbers. Saddle syndrome is due to adhesions of the lumbrical-interosseous intermuscular junctions. It is very often overlooked. In rheumatoid arthritis, tendon tenosynovitis of the IOM is frequently encountered. Enhancement of the LM may be an early imaging marker of the disease. Multiple tumor or pseudotumoral masses can develop within or around IMH. All of these pathologies can be accurately assessed using high-resolution dynamic ultrasound (US) and high-field 3T magnetic resonance imaging (MRI).

**Method:**

This educational review presents the aspects of the various IMH pathologies on high-resolution US and high-field 3T MRI.

**Results and Conclusion:**

There are numerous IMH pathologies. A radiologist’s knowledge and assessment of these various pathologies allows clinicians and surgeons to make an early and accurate diagnosis and choose the most appropriate treatment.

**Key Points:**

- High-resolution US and high-field 3T MRI allow for a precise assessment of various intrinsic hand muscle pathologies.
- Hand muscle denervation syndromes are common in median and ulnar nerve pathologies.
- Myositis of the intrinsic muscles of the hand occurs in a variety of settings.
- In rheumatoid arthritis, tenosynovitis of the intrinsic hand muscles is common, and enhancement of the lumbrical muscles may represent an early imaging marker.
- MRI facilitates early detection of saddle syndrome.

**Citation Format:**

- Bouredoucen H, Boudabbous S, Poletti P et al. Imaging of the intrinsic muscles of the hand – Part II: Pathological aspects on high-resolution ultrasound and 3 T MRI. Rofo 2025; DOI 10.1055/a-2735-2780

## Zusammenfassung

**Hintergrund:**

Die Pathologien der intrinsischen Handmuskulatur (IHM) sind vielfältig. Muskeldenervationssyndrome der Hand treten häufig bei Erkrankungen des Nervus medianus (NM) und Nervus ulnaris (NU) auf. Diese kommen im Rahmen des Karpaltunnelsyndroms, des Guyon-Kanal-Syndroms, traumatischer, tumoröser, entzündlicher oder proximaler Nervenschädigungen aufgrund zervikaler Radikulopathien oder Plexusbrachialisläsionen vor. Myositiden der IHM treten in verschiedenen klinischen Kontexten auf. Traumatische Muskelverletzungen der Hand sind selten; charakteristische Läsionen sind etwa die des dritten Interossusmuskels (IOM) oder der Lumbricalmuskeln (LM) bei Kletterern. Das Sattelsyndrom entsteht durch Adhäsionen an den intermuskularen Übergängen zwischen Lumbrical- und Interossusmuskeln und wird häufig übersehen. Bei rheumatoider Arthritis ist eine Tenosynovitis der IOM häufig, und die Kontrastmittelaufnahme der LM kann als früher bildgebender Marker dienen. Multiple tumoröse oder pseudotumorale Raumforderungen können innerhalb oder um die IHM entstehen. Alle diese Pathologien lassen sich hervorragend mittels hochauflösendem dynamischem Ultraschall (US) sowie hochfeldiger 3-Tesla-Magnetresonanztomografie (MRT) beurteilen.

**Methode:**

Diese Übersichtsarbeit stellt die Aspekte verschiedener Pathologien der IHM anhand von hochauflösendem Ultraschall und hochfeldiger 3-Tesla-MRT dar.

**Ergebnisse und Schlussfolgerung:**

Die Pathologien der intrinsischen Handmuskulatur sind zahlreich. Die Kenntnis und Beurteilung dieser Erkrankungen durch Radiologen ermöglicht eine frühzeitige und präzise Diagnosestellung und unterstützt Kliniker sowie Chirurgen bei der Auswahl der optimalen Therapie.

**Kernaussagen:**

- Hochauflösender Ultraschall und hochfeldige 3-Tesla-MRT erlauben eine präzise Beurteilung verschiedener Pathologien der intrinsischen Handmuskulatur.
- Denervationssyndrome der Handmuskulatur sind häufig bei Erkrankungen des Nervus medianus und Nervus ulnaris.
- Myositiden der intrinsischen Handmuskulatur treten in unterschiedlichen klinischen Kontexten auf.
- Bei rheumatoider Arthritis ist die Tenosynovitis der intrinsischen Handmuskulatur häufig, und die Kontrastmittelaufnahme der Lumbricalmuskeln kann ein früher bildgebender Marker sein.
- MRT erleichtert die frühzeitige Erkennung des Sattelsyndroms.

## Abbreviations

IMH: Intrinsic muscles of the hand
IOM: Interosseous muscle
LM: Lumbrical muscle
MN: Median nerve
MRI: Magnetic resonance imaging
PD FS: Proton-density fat-saturated
UN: Ulnar nerve
US: Ultrasound

## Introduction

Knowledge of the anatomy and anatomical variants of the IMH 1 [accessory abductor digiti minimi muscle (aADM), adductor hypothenar muscle, extensor digitorum brevis manus muscle (EDBM), variants of the LM, and the accessory flexor digitorum superficialis muscle of the index finger] is essential for recognizing asymptomatic or symptom-causing features, particularly in canal syndromes, carpal tunnel syndrome and Guyon’s canal syndrome, using high-resolution US and high-field 3T MRI. IMH have their own pathologies: hand muscle denervation syndromes, myositis, traumatic hand muscle injuries, interosseous tendons tenosynovitis, IOM involvement in Dupuytren’s contracture, and tumor and pseudotumoral lesions. Hand muscle denervation syndromes are common in pathologies of the MN and UN. They occur following carpal tunnel syndrome, Guyon’s canal syndrome, traumatic, tumoral, inflammatory, proximal nerve lesions due to upper cervical radiculopathy, or brachial plexus lesions. There are multiple causes of myositis. They include autoimmune diseases, myopathies, injuries, infections, drug side effects, fluid and electrolyte disorders, and idiopathic myositis. Traumatic muscle injuries of the hand usually occur following penetrating trauma, or rarely in the case of blunt injuries. Traumatic distraction injuries of the IOM are extremely rare. Saddle muscle syndrome manifests as pain in the distal intermetacarpal space when making a fist or during repetitive gripping activities. It is due to adhesions at the intermuscular junctions and can be caused by muscle anatomical variations. It is very often overlooked. The “quadriga effect” of LM injury is a classic injury in climbers. In rheumatoid arthritis, interosseous tendon tenosynovitis and LM enhancement are frequently found. The IOMs and their tendons can be affected in Dupuytren’s contracture. Tumor and pseudotumoral lesions can develop in or around the IMH. These various pathologies are diagnosed using high-resolution US and high-field 3T MRI. This educational review presents the imaging aspects of the various pathologies of IMH.

## Methodology

For this review, a systematic literature search was conducted to ensure a comprehensive and reproducible analysis of pathologies affecting the intrinsic muscles of the hand. The databases used in the search included PubMed and MEDLINE, covering all relevant articles published up to the end of March 2025. The search strategy combined English-language keywords used individually or in combination, including: “intrinsic hand muscles”, “thenar muscles”, “hypothenar muscles”, “lumbrical muscles”, “flexor digitorum superficialis variants”, “accessory hand muscles”, “carpal tunnel syndrome”, “Guyon’s canal syndrome”, “anatomical variations”, “muscle anomalies”, “hand ultrasonography”, “muscle denervation”, “myositis”, “compartment syndrome”, “hand trauma”, “hand tumors”, “saddle syndrome”, and “MR neurography”. Only articles published in English, available in full text, and addressing pathological aspects of the intrinsic hand muscles were included. Inclusion criteria encompassed original research articles, systematic reviews, meta-analyses, anatomical, imaging, or surgical studies, as well as clinical case reports discussing intrinsic muscle pathologies and their clinical implications. Articles not directly related to the topic, lacking relevant anatomical or clinical data, or not available in full text were excluded. This rigorous approach allowed a structured and targeted selection of the literature to support the scope and conclusions of this part of the review.

## Muscle Denervation

**Fig. 1:**
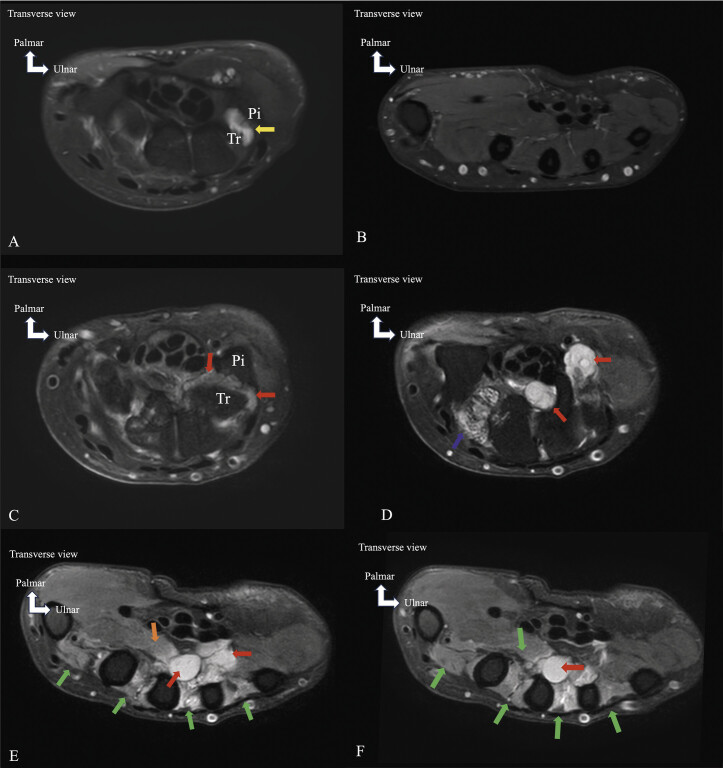
Non-compressive pisotriquetral arthrosynovial cyst ( **A** , **B** ) and compressive pisotriquetral arthrosynovial cyst with muscle denervation edema ( **C** , **D** , **E** , **F** ). Axial proton-density fat-saturated (PD FS) MRI images. A small pisotriquetral arthrosynovial cyst ( **A** ) (white arrow) is seen without UN compression, as indicated by the absence of muscle edema in muscles innervated by the UN ( **B** ). In contrast, a large cyst ( **C** , **D** , **E** , **F** ) extends palmarly, behind the carpal tunnel, and dorsally into the interosseous space, compressing the UN in Guyon’s canal and its terminal branches. Muscle denervation edema affects the muscles innervated by the deep motor branch of the UN, involving all ventral and dorsal interosseous muscles ( **E** , **F** ) (green arrows) except the first ventral interosseous muscle, as well as the adductor pollicis muscle ( **E** ) (orange arrow). Edema of the trapezoid related to arthropathy is also present ( **D** ) (blue arrow). Pi = pisiform, Tr = triquetrum.

**Fig. 2:**
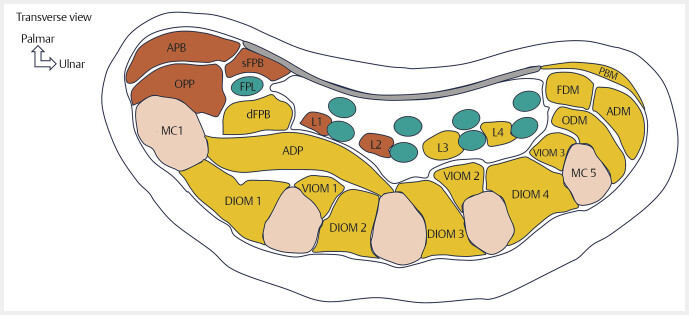
Schematic drawings showing the intrinsic muscles of the hand and their innervations in a transverse view. Muscles innervated by the median nerve (in orange). Muscles innervated by the ulnar nerve (in yellow). Thenar muscles: Abductor pollicis brevis (APB), opponens pollicis (OPP), deep flexor pollicis brevis (dFPB), superficial flexor pollicis brevis (sFPB), adductor pollicis (ADP). Hypothenar muscles: Palmaris brevis muscle (PBM), flexor digiti minimi (FDM), opponens digiti minimi (ODM), abductor digiti minimi (ADM). Lumbrical muscles: first lumbrical muscle (L1), second lumbrical muscle (L2), third lumbrical muscle (L3), fourth lumbrical muscle (L4). Interosseous muscles: first ventral interosseous muscle (VIOM 1), second ventral interosseous muscle (VIOM 2), third ventral interosseous muscle (VIOM 3), first dorsal interosseous muscle (DIOM 1), second dorsal interosseous muscle (DIOM 2), third dorsal interosseous muscle (DIOM 3), fourth dorsal interosseous muscle (DIOM 4).

Denervation refers to changes experienced by the muscle following the partial or complete loss of its innervation. Chronologically, edema, atrophy, and later, fatty muscle degeneration appear 2 . MRI allows for a positive diagnosis of denervation ( Fig. 1 ). In addition to signal abnormalities, systematization within a single innervation territory is a fundamental element that allows for a topographic diagnosis of the muscle and, consequently, the altered nerve responsible for the muscle denervation (the innervation territories of the MN and UN). Some muscle variants benefit from dual innervation and constitute diagnostic pitfalls. These must, therefore, be recognized. In the acute phase (<1 month), MRI shows an early and homogeneous muscle STIR (Short tau Inversion Recovery) hyperintensity without abnormalities on T1-weighted images. This hyperintensity is observed after the first 24 hours in animals and after the fourth day in clinical situations 3 . In the subacute phase (1 month to 1 year), muscle edema persists, most often for less than 10 weeks and in exceptional cases for more than six months. Early muscle atrophy is also seen 4 . In the acute and subacute phases, homogeneous contrast enhancement of the denervated muscle is observed, which should not be misinterpreted as an inflammatory or tumoral lesion 5 . In the chronic phase (over 1 year), muscle edema disappears. T1-weighted imaging can quantify muscle atrophy and fatty degeneration 6 . US has difficulty detecting early muscle denervation edema as hypoechoic infiltration of the affected muscles. However, it more easily detects fatty infiltration as a hyperechoic appearance of the examined muscle. Muscle denervation syndromes of the hand are common. The two nerves that innervate the IMH are the MN and the UN ( Fig. 2 ). There are multiple MN pathologies, with carpal tunnel syndrome being the most common compressive syndrome with an estimated prevalence of 4–5% 7 , making it the most common neuropathy of the wrist and hand. It may be due to acute neuropathy, trauma (transection, acute stretching, and compression), fracture, especially radial, or midcarpal instability. It may result from systemic pathologies, such as amyloidosis, rheumatoid arthritis, intrinsic compressive lesions of the MN within the carpal tunnel, or extrinsic lesions in the adjacent environment of the carpal tunnel, such as tenosynovitis, cyst, hematoma, fracture callus, muscle variants, nerve sheath tumors, or malignant tumor lesions 8 . Muscle denervation is an ancillary sign in moderate to severe cases of carpal tunnel syndrome. The most common hand denervation syndrome occurs in carpal tunnel syndrome, causing atrophy of the muscles innervated by the MN ( Fig. 2 ), the thenar muscles (abductor pollicis brevis, superficial flexor pollicis brevis, and opponens pollicis); and the first and second LM 9 . After carpal tunnel release surgery, ultrasound shows a progressive decrease in the cross-sectional area of the median nerve, which correlates with clinical improvement. An average reduction of up to 1.7 mm² at six months has been reported 10 11 . On MRI, an initial increase in nerve and carpal tunnel volume reflects decompression, followed by reorganization of the retinaculum within one month 12 13 . At three months, nerve flattening and swelling often persist without signs of active compression. Between six and twelve months, a significant reduction in proximal nerve volume and T2 signal indicates resolution of inflammatory edema 14 15 . Residual morphological abnormalities may remain without clinical signs of recurrence 13 16 . Thumb opposition recovery after surgery for severe carpal tunnel syndrome was evaluated in a pilot study combining clinical, radiological, and electrophysiological approaches. In patients with thenar muscle atrophy, surgical release of the carpal tunnel resulted in significant functional improvement, notably in thumb opposition and pinch strength. Electrophysiology showed improved muscle potential of the abductor pollicis muscle. MR neurography revealed significant improvement in the morphology of the thenar branch of the median nerve and a marked decrease in denervation signs, while the overall morphology of the median nerve showed no significant change at six months. These results support the value of a multidimensional assessment to predict and monitor functional recovery after surgery 16 .

**Fig. 3:**
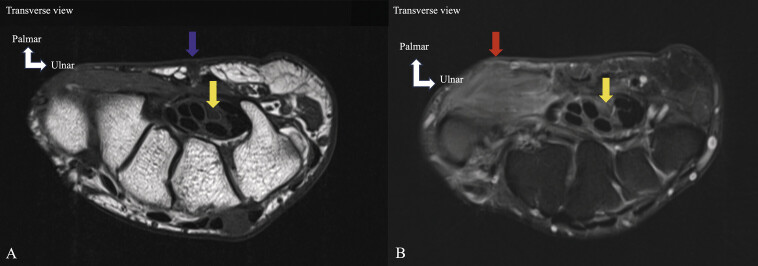
Recurrent carpal tunnel syndrome. MRI ( **A** , **B** ) on axial T1 TSE ( **A** ) and PD FS ( **B** ). MRI performed several weeks after surgical release, edema of the thenar muscles (abductor pollicis brevis, opponens pollicis, and flexor pollicis brevis) ( **B** ) (red arrow), loss of fibrillar appearance and edema of the MN ( **A** , **B** ) (yellow arrows). The MRI appearance suggests ongoing neuropathy. Scar (A) (blue arrow). Denervation may reflect iatrogenic injury to the MN.

Recurrent carpal tunnel syndrome is characterized by the return of symptoms after at least a three-month symptom-free interval following surgical release. This recurrence, often after more than six months of relief, suggests incomplete decompression or complications such as perineural adhesions, scar fibrosis, intraneural fibrosis, reformation of the transverse ligament, persistent tenosynovitis, or the presence of masses 17 18 . In recurrent cases, dynamic ultrasound identifies areas of persistent compression and scar tissue limiting nerve mobility 19 . On MRI, moderate to marked signal changes in the nerve and innervated muscles persisting six weeks after release suggest ongoing neuropathy ( Fig. 3 ) 8 . MRI demonstrates fibrosis, nerve flattening and enlargement, and tenosynovitis, and detects deep fibrosis, thus facilitating surgical planning. However, some criteria such as T2 signal or skin-to-nerve distance lack specificity 20 21 . Diffusion tensor imaging (DTI) reveals microstructural alterations related to endoneural fibrosis and functional impairment, making it a promising tool for follow-up 22 . MR neurography assesses persistent neuropathy and detects iatrogenic lesions, perineural fibrosis, and recurrent nerve compression 8 23 . A prolonged decrease in fractional anisotropy confirms evolving neuropathy 24 . Complications such as tenosynovitis, hematoma, or ganglion cysts may delay recovery 8 . The American Academy of Orthopedic Surgeons has published guidelines for the surgical management of primary carpal tunnel syndrome, but there are no specific recommendations for recurrent carpal tunnel syndrome. Surgical revision in these cases, therefore, relies on clinical, electrophysiological, and radiological evaluation 18 25 .

**Fig. 4:**
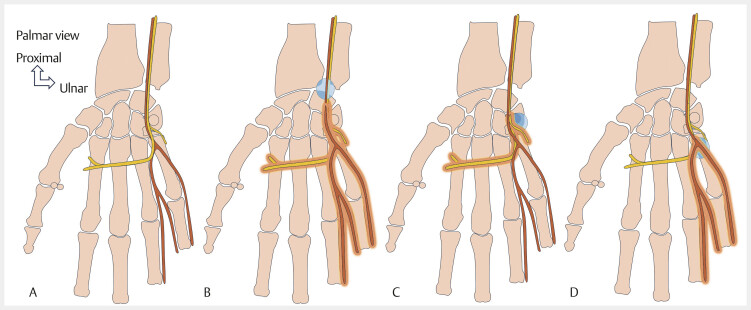
Schematic drawing showing the areas of ulnar nerve compression in Guyon’s canal. The sensory branch of the ulnar nerve is shown in brown, and the deep motor branch of the ulnar nerve is shown in yellow ( **A** ). In Guyon’s canal, there are three types of ulnar nerve compression zones, based on the anatomical course of the nerve. Zone I compression ( **B** ) refers to proximal or intracanal compression before the ulnar nerve bifurcates into superficial and deep branches. It transmits the sensory and motor bundles of the ulnar nerve. Compression in this zone manifests as motor weakness in all muscles innervated by the ulnar nerve, and sensory deficits in the hypothenar eminence, fifth, and fourth fingers. Zone II ( **C** ) compression exclusively affects the motor branch distal to the bifurcation of the NU. It manifests as motor weakness of the muscles innervated by the NU, with intact sensation along the distribution of the NU. Zone III ( **D** ) compression results from compression of the sensory branch and, therefore, manifests as sensory loss without muscle weakness.

**Fig. 5:**
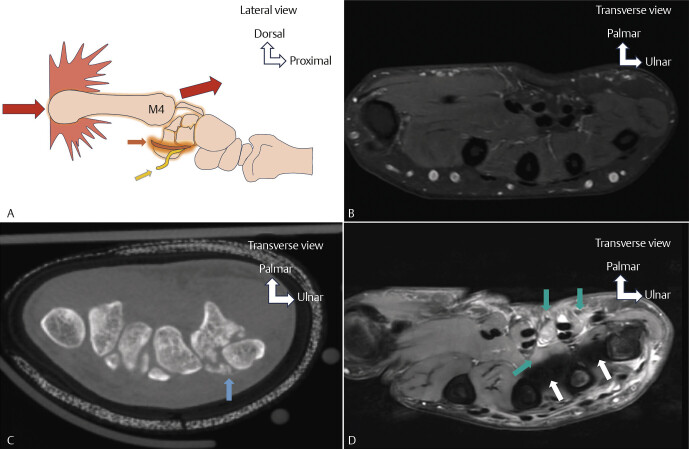
Contusion injury of the deep motor branch of the UN following a carpometacarpal dislocation with fracture-impaction of the hamate ( **A** , **C** , **D** ). Normal axial PD FS MRI showing normal muscle signal without denervation edema ( **B** ). Schematic drawing ( **A** ) illustrating the injury mechanism: a missed punch causes the fist to deviate and land on the fourth and fifth metacarpals, which are mobile. Longitudinal compression along the metacarpal axis with proximal and dorsal displacement of the metacarpal transmits force to the base of the fourth metacarpal (M4), causing a fracture and impaction of the hamate with direct and severe compression of the deep motor branch of the UN. Deep motor branch of the UN ( **A** ) (orange arrow), sensory branch of the UN ( **A** ) (yellow arrow). CBCT axial reconstruction ( **C** ) shows a comminuted fracture and impaction of the hamate (blue arrow). Axial PD FS MRI ( **D** ) reveals denervation edema of the interosseous and lumbrical muscles (green arrows). Artifact from osteosynthesis hardware (white arrows).

Denervation may indicate an iatrogenic injury. Muscle denervation is sought in other MN pathologies, blunt or penetrating trauma to the MN, common benign tumor lesions of the MN that include schwannoma and neurofibroma or malignant nerve sheath tumors, diffuse neuropathy [genetic, autoimmune, and inflammatory neuropathies, such as Charcot-Marie-Tooth (CMT) disease, chronic inflammatory demyelinating polyneuropathy (CIDP), and multifocal motor neuropathy (MMN)] 26 27 , or proximal involvement due to upper cervical radiculopathy, brachial plexus lesions, or pronator syndrome, which can present with muscle denervation in the forearm and hand 8 . UN pathologies occur in various situations. Guyon’s canal syndrome is the second most common denervation syndrome. It results from compression of the UN in the Guyon’s canal. Symptoms can vary, ranging from mixed motor-sensory, purely motor, or purely sensory, depending on the site of entrapment, from proximal to distal, respectively ( Fig. 4 ). This entrapment neuropathy in the Guyon’s canal often has a primary cause, unlike carpal tunnel syndrome which is mainly idiopathic. It can originate from a cyst, a lipoma, a hematoma, an accessory abductor digiti minimi 1 , a fracture in particular of the hamulus of the hamate, postoperative scarring, thickened fascia, occlusion of the ulnar artery or aneurysm (hypothenar hammer syndrome), thrombosis of the ulnar vein, tumors 8 , or microtraumatic injury to the UN encountered in the context of so-called cyclist’s handlebar palsy 28 . In ulnar neuropathy, secondary signs of denervation are located in the muscles innervated by the UN ( Fig. 2 ), the hypothenar muscles (abductor digiti minimi, flexor digiti minimi, opponens digiti minimi), the third and fourth LM, the adductor pollicis, the deep flexor pollicis brevis, and all ventral and dorsal IOMs, particularly the first dorsal IOM, the latter serving as a terminal muscle along the entire course of the UN 29 . Isolated neuropathy of the deep motor branch of the UN can be detected ( Fig. 5 ), causing muscle denervation, signal abnormalities of the deep motor branch of the UN at the metacarpals, with normal appearance of the nerve in the Guyon’s canal, and its superficial branch 8 . Partial involvement of the hypothenar muscles can occur in nerve compressions located in a terminal nerve branch of a specific muscle. Selective muscle denervation reflects the corresponding nerve damage. The cysts typically originate from the pisotriquetral joint, impinging on the main trunk of the UN, but they can also arise from the triquetrum-hamate and carpometacarpal joints. In the latter cases, they selectively compress the deep motor branch of the UN at different points along its course through the palm. Like the MN, the UN can also be affected by diffuse neuropathy or proximal neuropathy 8 .

Recommendations regarding the diagnosis and management of muscle denervation primarily rely on MRI, which allows early detection of edema, followed by atrophy and fatty degeneration, enabling precise diagnosis according to the affected nerve territory 2 3 4 5 6 . Ultrasound is less effective in the acute phase but is useful for detecting fatty infiltration. For median nerve pathologies, particularly carpal tunnel syndrome, MRI is recommended in cases of recurrence to assess persistent neuropathy 7 8 9 . Regarding the ulnar nerve, MRI identifies compressions in Guyon’s canal and their various causes 8 28 29 . These recommendations are based on strong experimental and clinical studies, except for in the case of some rare causes where they rely on case reports, limiting the level of evidence.

## Myositis

**Fig. 6:**
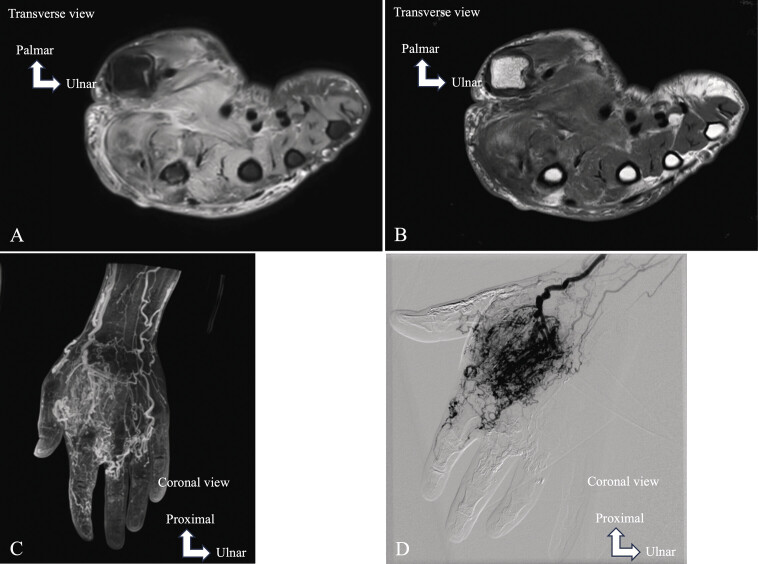
Chronic myositis due to an arteriovenous malformation of the thenar eminence and first commissure. MRI on axial PD FS ( **A** ) and axial T1 TSE ( **B** ). MR angiography ( **C** ). Conventional angiography ( **D** ). Diffuse muscle edema with hypersignal on PD FS due to myositis ( **A** ). Fatty involution of the muscles with hypersignal on T1 TSE due to chronic myositis ( **B** ). MR angiography ( **C** ) and conventional angiography ( **A** ) were performed to analyze the vascular branches supplying this arteriovenous malformation.

The term myositis refers to an inflammatory infiltrate of striated muscles. There are multiple causes of myositis including autoimmune diseases, myopathies, injuries, infections, drug side effects, fluid and electrolyte disorders, and idiopathic myositis. MRI shows increased signal intensity on T2-weighted (fat-suppressed) images, as well as contrast enhancement on T1-weighted (fat-suppressed) MRI images ( Fig. 6 ). MRI is the only imaging modality that explores myositis. It reveals muscle edema, usually multicompartmental. A key feature that differentiates it from muscle denervation edema is the absence of distribution of the edema in an innervated area. Understanding the clinical context is essential to differentiate the causes of myositis, as its MRI appearance is often nonspecific. Infectious hand myositis can be acute or chronic, with staphylococcus aureus being the most common pathogen. Radiological findings can suggest the infectious agent. Bacterial myositis manifests as focal muscle involvement, while more diffuse involvement is more likely to be viral or parasitic myositis. Infections are generally related to penetrating skin lesions, trauma, or animal bites to the hand. Infections can occur following surgery. MRI scans look for abscesses in the form of fluid accumulation (high T2 signal, low T1 signal) with peripheral shell enhancement after contrast injection.

MRI is the imaging modality of choice for evaluating myositis, as it effectively detects muscle edema and potential infectious collections 2 . These recommendations are based on solid clinical data. However, certain infectious or atypical presentations rely primarily on isolated case reports, which limits the overall level of evidence 2 .

## Hand Compartment Syndrome

**Fig. 7:**
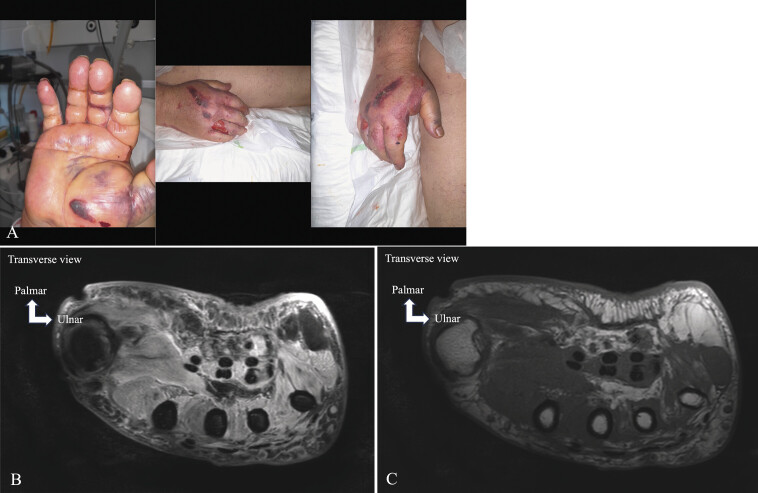
Acute compartment syndrome and rhabdomyolysis. Fall and stationary position on the ground for several hours. Dermabrasion and swelling of the hand ( **A** ). PD FS MRI on axial ( **B** ), diffuse edema with foci of muscle necrosis of the IMH. T1 TSE MRI on axial ( **C** ), disorganization of the muscle structures of the IMH.

Acute compartment syndrome of the hand is rare. It has multiple etiologies, including traumatic (fractures, dislocations, blunt trauma, crush injuries, penetrating/gunshot injuries, burns, envenomation, high-pressure injection), medical (infection, bleeding disorders, spontaneous hemorrhage, rhabdomyolysis), iatrogenic (ischemia-reperfusion, intravenous infiltration, contrast extravasation, constrictive dressings or casts, arthroscopy, prolonged pressure due to positioning), or vascular (arterial injuries, arterial puncture, arterial catheterization, or venous occlusion) 30 . Rhabdomyolysis is a necrotizing complication. It usually develops rapidly and can be complicated by compartment syndrome due to the rapid increase in pressure in the muscle compartments between the fascia. MRI shows intramuscular hemorrhage ( Fig. 7 ). Depending on the severity of the case and without early and appropriate treatment, the outcome can range from minimal functional deficits to limb loss or even death. The resulting pressure fluctuations, extravasation of intravenous fluids, and ischemia are subject to debate. Nonspecific and likely unreliable and inaccurate criteria are used to support a diagnosis of hand compartment syndrome: the extent of swelling, compartment tension on palpation, and pain intensity 31 . According to Halpern et al., the most sensitive clinical sign of hand compartment syndrome is pain upon passive mobilization at the metacarpophalangeal joint corresponding to the affected intrinsic musculature 32 . Objective criteria to aid in diagnosis are limited. Currently, there is no consensus reference for the diagnosis of hand compartment syndrome 31 . Some surgeons rely on objective measurement of intracompartmental pressure when the diagnosis of compartment syndrome is uncertain 31 , but the threshold pressure for the diagnosis of compartment syndrome of the hand is under debate, and there is currently no consensus regarding the absolute pressure or pressure difference between measured intracompartmental pressure and diastolic blood pressure (delta P) that clearly diagnoses compartment syndrome of the hand 31 . Furthermore, there is variability in the compartmentalization of the myofascial spaces of the hand 33 , and the clinical effect of this subcompartmentalization on the measurement of compartment pressures and the treatment of hand compartment syndrome is unknown. Based on the general understanding of altered tissue metabolism and muscle ischemia at this pressure, Codding et al. recommend releasing the hand compartments when pressures are within 30 mm Hg of the patient’s diastolic blood pressure (delta P < 30) 31 .

Chronic exertional compartment syndrome of the hand is also rare 34 . Clinically, it is characterized by acute pain, cramps, and significant swelling, induced by physical activity or repetitive grasping and twisting of the hand, and requires periods of rest for relief 34 . Provocative maneuvers most often included repetitive pinching. Pressure is measured immediately after the onset of symptoms and 5 minutes after rest when symptoms have resolved. The different compartments of the hand are variably affected 34 . Differential diagnoses may include arthritis, nerve compression, and focal dystonia (writer’s cramp) 34 . Surgical treatment consists of releasing the affected compartments.

Recommendations for the diagnosis and management of hand compartment syndrome remain limited due to the lack of consensus criteria and standardized pressure thresholds. While some advocate surgical decompression when intracompartmental pressure is within 30 mm Hg of diastolic blood pressure (delta P < 30) 31 , such thresholds are mainly based on expert opinion and limited clinical case series. Overall, current recommendations rely on observational data, clinical expertise, and isolated case reports 30 31 32 33 34 , reflecting a moderate to low level of evidence given the rarity and variability of the condition.

## Intrinsic Muscle Trauma

**Fig. 8:**
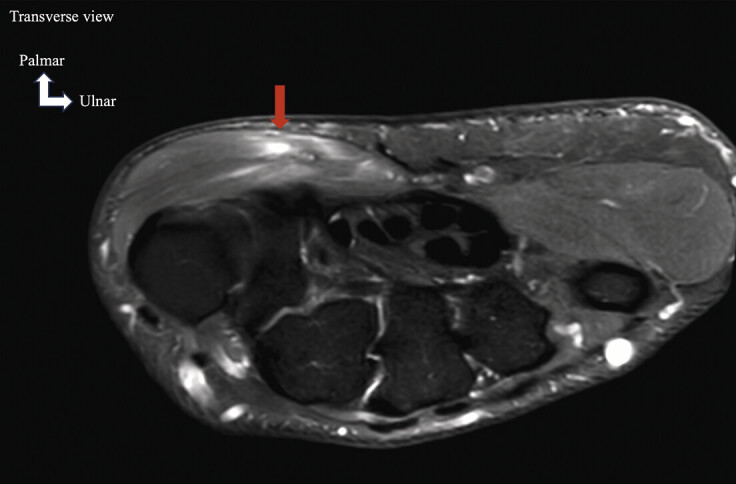
Contusion of the thenar muscles during direct trauma. Hand pain following a fall with landing on the thenar eminence. Suspected scaphoid fracture. Axial FS PD MRI shows a thenar muscle injury with significant edema (red arrow).

**Fig. 9:**
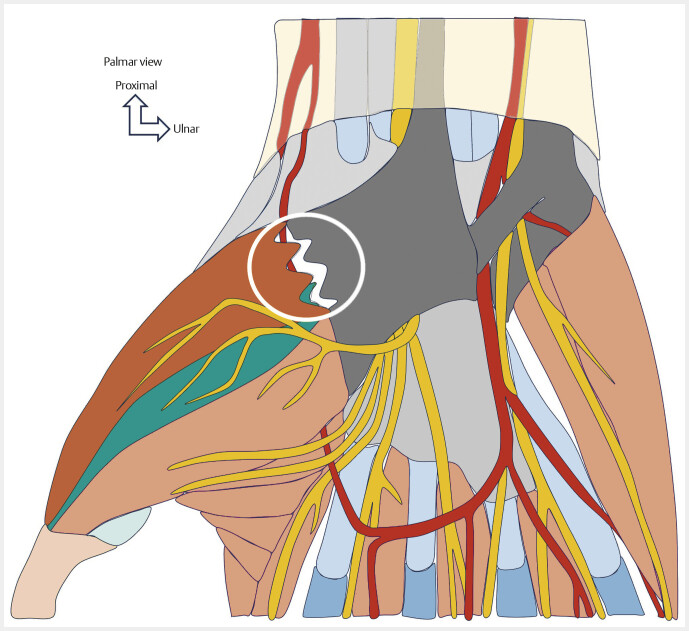
Schematic drawing showing complete tendon avulsion of the thenar muscles (area circled in white). Avulsion of the APB from its insertions on the anterior surface of the transverse carpal ligament, the scaphoid tubercle, or avulsion of the OPP from its insertions on the carpometacarpal joint capsule, the trapezius tubercle, and the anterior surface of the transverse carpal ligament are rare. Surgical treatment is recommended.

**Fig. 10:**
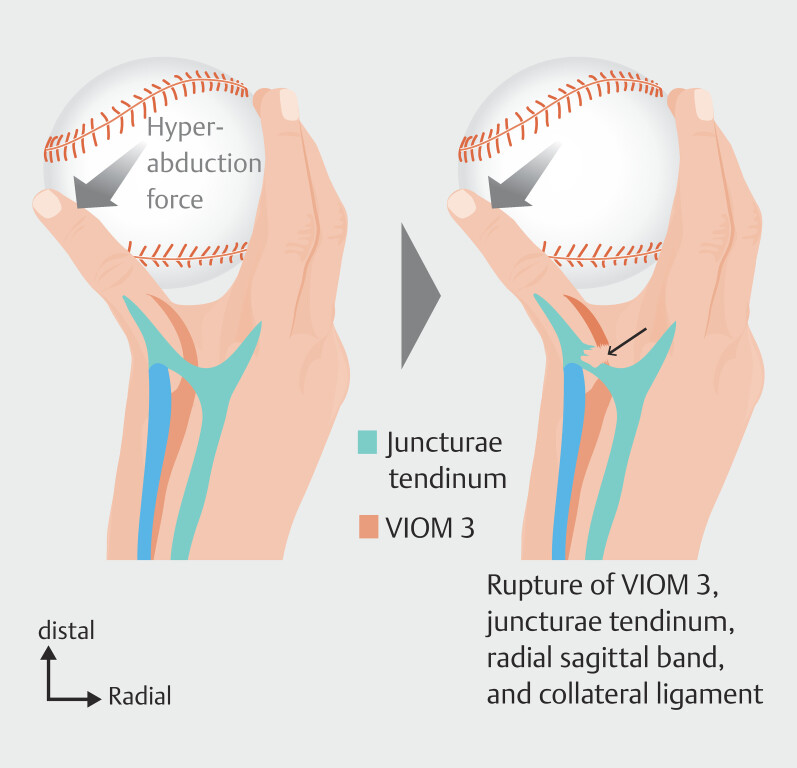
Schematic drawing showing the pathoanatomy of third ventral interosseous muscle (VIOM 3) rupture. The mechanism is forced hyperabduction, as in this example in baseball players, resulting in ruptures of VIOM 3, the juncturae tendinum, the radial sagittal band, and the radial collateral ligament. The figure is based on: Lourie GM, Lundy DW, Rudolph HP, Bayne LG. Abducted, hyperextended small finger deformity of nonneurologic etiology. J Hand Surg Am. 1999 Mar;24(2):315–319.

**Fig. 11:**
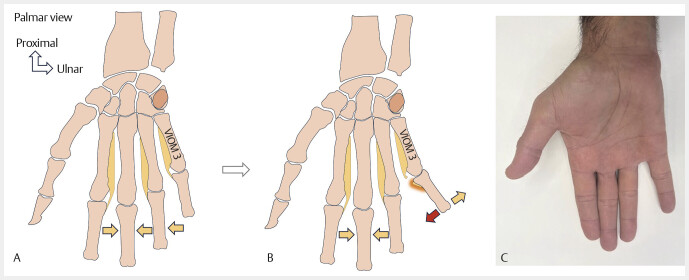
VIOM 3 is the only muscle that adducts the 5th finger ( **A** ). Its rupture causes an abduction deformity ( **B** , **C** ).

**Fig. 12:**
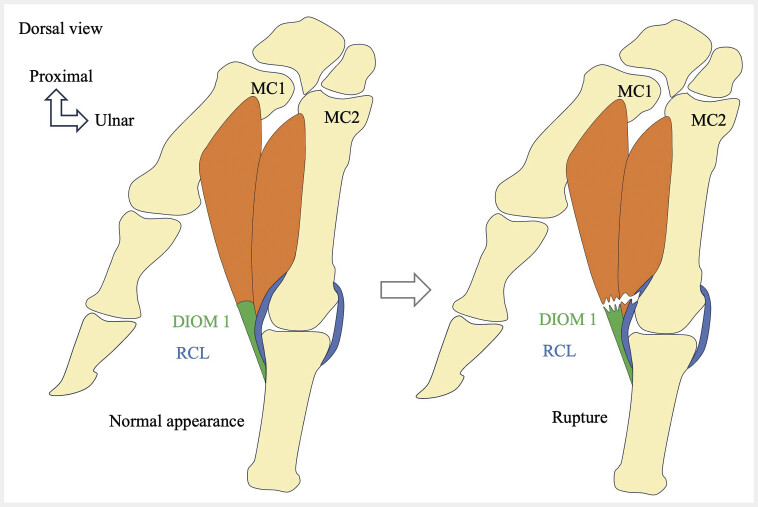
Schematic drawing showing a distal traumatic rupture of the tendon of the first dorsal interosseous muscle (DIOM 1), with extension of the lesion into the radial collateral ligament (RCL) of the second metacarpophalangeal joint.

Traumatic muscle injuries of the hand and wrist usually occur following penetrating trauma. Blunt traumatic injuries of the thenar muscles are rare. In muscle contusions, MRI shows muscle edema, which is distinguished from denervation edema by the lack of systematization in the same innervation area ( Fig. 8 ). Traumatic injuries can cause complete avulsion of the tendinous origins of the abductor pollicis brevis and opponens pollicis ( Fig. 9 ), producing pain and weakness in thumb abduction and opposition 35 . Distraction injuries of the IOMs are extremely rare and are sometimes associated with lesions of the LM tendons or collateral ligaments of the metacarpophalangeal joint ( Fig. 10 ). Clinically, an inability to abduct/adduct the finger should lead to the search for these lesions, as they may require surgical treatment. A lesion of the third ventral IOM may cause difficulties in adduction and flexion of the fifth finger, as the third ventral IOM is the only muscle that contributes to adduction of the little finger ( Fig. 11 ) 36 37 . US can recognize distraction injuries of the IOMs, distinguish low-grade muscle strain from tendon avulsion, and identify associated injuries involving locoregional structures ( Fig. 12 ).

The diagnostic and therapeutic strategy for intrinsic muscle trauma of the hand primarily relies on MRI, which is effective for identifying contusions, tendon avulsions, and deep muscle injuries, while distinguishing traumatic edema from denervation-related changes 35 36 37 . US plays a complementary role in evaluating interosseous muscle distraction injuries, detecting associated tendon damage, and supporting surgical planning when required 36 . This approach is grounded in clinical case series and observational reports, with limited high-level evidence due to the rarity of these injuries.

## Saddle Syndrome (Lumbrical-Interosseous Syndrome)

The lumbrical muscles cross palmarly to the deep transverse metacarpal ligament, while the dorsal and palmar interosseous muscles cross dorsally. The confluence of these two tendons, known as the interosseous-lumbrical junction, forms a “saddle” that moves proximally toward the deep transverse metacarpal ligament upon activation of the intrinsic muscles.

**Fig. 13:**
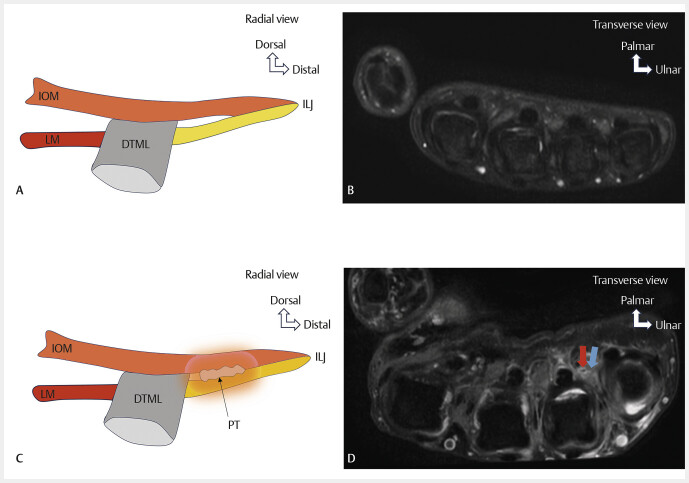
Normal aspect of the lumbrical-interosseous junction ( **A** , **B** ), saddle syndrome ( **C** , **D** ). Schematic drawing ( **A** , **C** ). Axial proton-density fat-saturated (PD FS) MRI images ( **B** , **D** ). In the absence of saddle syndrome ( **A** ), no abnormal signal is observed in the space between the lumbrical-interosseous junction and the deep transverse metacarpal ligament ( **B** ). Schematic drawing showing the pathoanatomy of saddle syndrome ( **C** ). The lumbrical muscles cross palmarly to the ligament, while the ventral and dorsal interosseous muscles pass dorsally. Adhesions or scar tissue (pseudotendon: PT) are present in the space between the lumbrical-interosseous junction and the ligament. During intrinsic muscle activation, impingement occurs between the inflamed sella and the ligament. Saddle syndrome on MRI ( **D** ), axial PD FS sequence, showing scarring and inflammatory reaction of the lumbrical-interosseous muscles and tendons, with no clear distinction between lumbrical and interosseous tendons and the ligament (blue arrow). Lumbrical tendon (red arrow).

Saddle syndrome is a rare condition. Its presumed pathology is the formation of adhesions or scar tissue in the space between the interosseous-lumbrical junction and the deep transverse metacarpal ligament ( Fig. 13 ). These most often occur in a post-traumatic setting. During intrinsic activation, impingement occurs between the deep transverse metacarpal ligament and the inflamed sella 38 . The anatomical variants of the IMH (morphological and insertion points along the first phalanx) could result in a predisposition to adhesions 39 40 41 . Patients present with pain in the distal intermetacarpal space. The diagnosis is often overlooked. MRI assists in the diagnosis of saddle joint deformity by identifying localized edema and scar tissue and can help differentiate it from isolated intrinsic tension 42 43 .

Assessment and management of saddle syndrome mainly rely on MRI, which allows detection of localized edema and scar adhesions at the interosseous-lumbrical junction, thus helping to differentiate this condition from isolated intrinsic muscle tension 42 43 . This rare and often overlooked syndrome requires imaging for accurate diagnosis, particularly after trauma. Evidence is largely based on case reports, thus limiting the strength of recommendations.

## Specific Lumbrical Muscle Pathologies

LM injuries were reported as a rare climbing-specific injury by Schweizer et al. 44 . In recent years, an increasing number of these injuries have been reported in patients as well as in elite international climbers 45 . Lesions typically occur at the third or fourth LM, as these are the only ones with a bipennate origin. These injuries are related to the “quadriga effect”. Gripping positions in which one or two fingers are extended while the neighboring fingers are flexed by force increase the maximum force by up to 50% and cause displacement of the flexor digitorum profundus tendons. Due to the dual origin of the bipennate lumbrical muscle body from the adjacent flexor digitorum profundus tendons, displacement of the flexor digitorum profundus tendons of the different fingers against each other leads to shear stress of the LM body and muscle damage. Treatment is conservative 44 45 46 . In the case of rheumatoid arthritis, lumbrical muscle enhancement could serve as a significant diagnostic marker of the disease in patients with suspected inflammatory joint disorders and unclear clinical and laboratory manifestations in the early stage of the disease 47 . Rheumatoid arthritis patients had a higher frequency of lumbrical muscle enhancement (8.5% high enhancement, 18.6% low enhancement), followed by other types of arthritis (3.6% high enhancement, 11.6% low enhancement) and control groups (0% high enhancement, 6.25% low enhancement) 47 . Damage to the first and second LM is observed in MN neuropathies, and to the third and fourth LMs in UN neuropathies. However, selective denervation of a single LM with preservation of the other muscles can occur following traumatic nerve injuries affecting distal motor branches or muscle injury 48 .

The diagnostic and therapeutic approach for specific lumbrical muscle pathologies primarily relies on thorough clinical evaluation combined with imaging, particularly MRI, which helps identify muscle injuries related to trauma or inflammatory conditions such as rheumatoid arthritis 44 45 46 47 48 . Treatment of traumatic injuries is generally conservative. These guidelines are based on clinical studies and case reports, with limited high-level evidence due to the rarity of these conditions.

## Specific Interosseous Muscle Pathologies

**Fig. 14:**
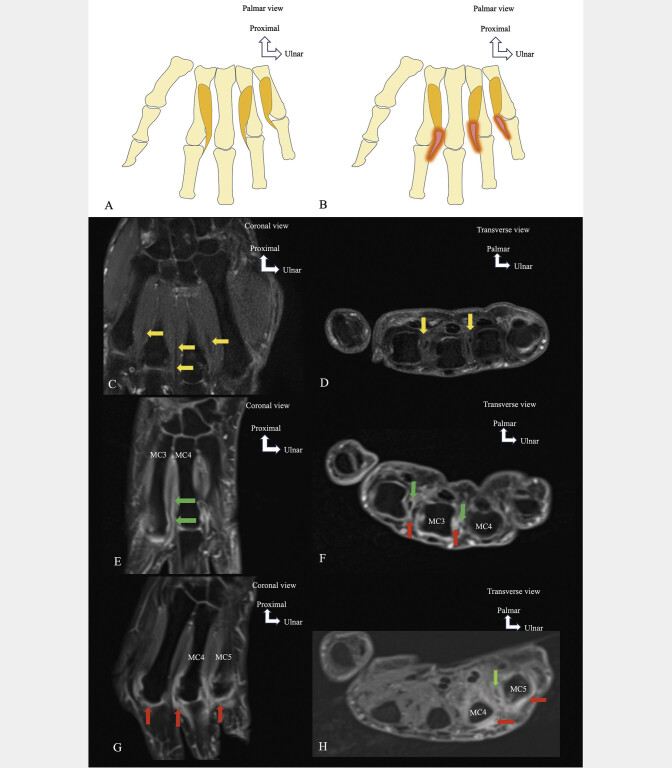
Schematic drawings showing the normal appearance of the interosseous muscle tendons ( **A** ) and tenosynovitis of the interosseous muscles in rheumatoid arthritis ( **B** ). Post-gadolinium T1-weighted fat-saturated MRI, coronal planes ( **C** , **E** , **G** ) and axial planes ( **D** , **F** , **H** ). Normal appearance of the interosseous muscle tendons without contrast enhancement ( **C** , **D** ) (yellow arrows). Contrast enhancement indicating tenosynovitis of the interosseous muscle tendons ( **E** , **F** , **H** ) (green arrows), adjacent to synovitis of the metacarpophalangeal joints ( **F** , **G** , **H** ) (red arrows).

In rheumatoid arthritis, tenosynovitis of the interosseous tendons of the hand was observed in 47.7% of rheumatoid arthritis patients ( Fig. 14 ). It was often adjacent to synovitis of the metacarpophalangeal joint, but it can be observed in isolation 49 . The interosseous tendons and their tendons can be affected in Dupuytren’s contracture 50 .

Diagnosis of interosseous muscle pathologies relies on MRI in rheumatoid arthritis 49 and surgical observations in Dupuytren’s disease 50 . These recommendations are based on descriptive data with limited high-level evidence.

## Tumors

Tumors and tumor-like lesions can develop within or around the IMH. Hand masses are often benign lesions 51 . Tumor-like lesions can present as ganglion cysts, accessory muscles, foreign body granulomas, tenosynovitis, epidermoid inclusion cysts, gout, rheumatoid nodules, or vascular lesions. The dorsal wrist is the most common location for cysts, and they are more common in women, most often encountered in the second to fourth decades of life. Palmar radial cysts are most common in the fifth to seventh decades of life 52 . On US, a cyst appears as a thin-walled anechoic or hypoechoic mass, sometimes with echogenic foci or internal septa, with posterior acoustic enhancement, and no internal vessels on Doppler examination. A search for a pedicle with the underlying joint should be performed 53 . On MRI, a cyst appears as a fluid-filled mass with minimal or no capsular enhancement 54 , near a joint or tendon sheath. An iso- or hyperintense signal from the cyst on T1-weighted images indicates hemorrhagic or protein content. The most common vascular lesions of the upper extremity are hemangiomas and vascular malformations 52 . Foreign body granulomas are a tissue reaction to retained foreign bodies after penetrating trauma. Localizing the foreign body can be difficult, particularly in patients with nonspecific symptoms and no history of a traumatic incident. Wood splinters are the most common foreign body in the hand after trauma 52 55 .

**Fig. 15:**
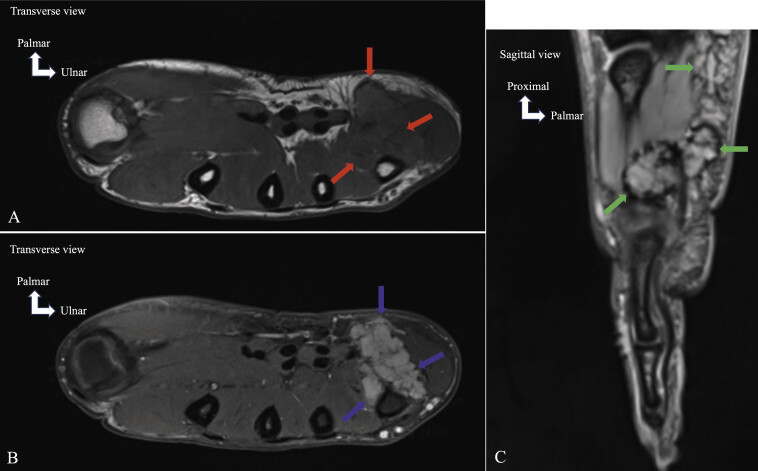
Tenosynovial giant cell tumor. MRI on axial T1-weighted ( **A** ), axial proton-density fat-saturated (PD FS) ( **B** ), and sagittal gradient-echo ( **C** ) planes. The T1-weighted image ( **A** ) demonstrates a heterogeneous mass with an intermediate/low signal (red arrows). The PD FS image ( **B** ) shows a heterogeneous low signal lesion (blue arrows). The gradient-echo image ( **C** ) reveals areas of low signal indicating the presence of hemosiderin (green arrows). Blooming effect, which refers to the local exaggeration of low signal due to hemosiderin, is seen on this sequence and serves as a key feature to differentiate TSGCT from inflammatory synovitis, which typically lacks this effect.

**Fig. 16:**
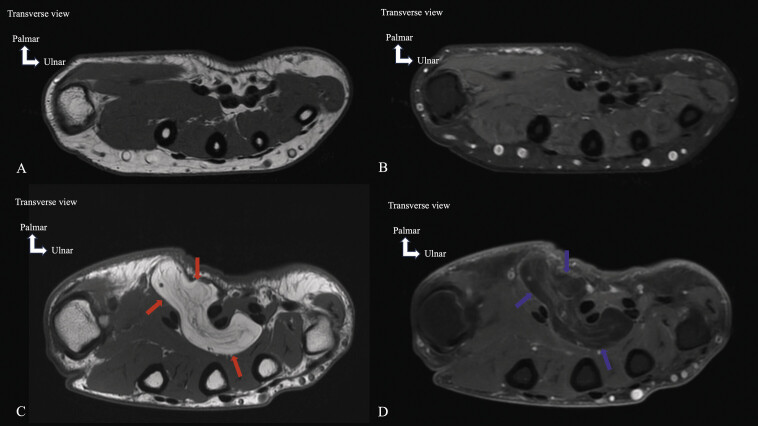
Lipoma. Magnetic resonance imaging in axial T1-weighted ( **A** , **C** ) and axial T1-weighted fat-saturated post-gadolinium ( **B** , **D** ) planes. The normal appearance of the compartment of the flexor pollicis longus muscle ( **A** , **B** ) and Lipoma ( **C** , **D** ). The T1 image ( **C** ) shows a high T1 signal mass (red arrows) with some internal streaks in the compartment of the flexor pollicis longus muscle. The axial T1 fat-saturated post-gadolinium image ( **D** ) reveals a homogeneous low-signal mass without enhancement (blue arrows). Despite the large size of this lesion, its homogeneous appearance, absence of adjacent soft tissue edema, absence of thick intralesional septa, and lack of contrast enhancement are in favor of benignity.

**Fig. 17:**
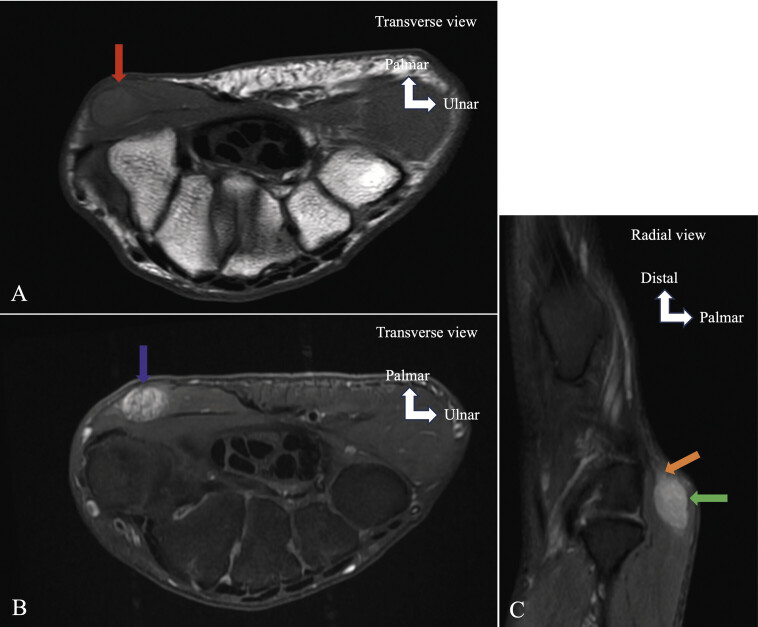
Schwannoma. Axial T1-weighted image **A** demonstrating an intermediate signal mass (red arrow) in the abductor pollicis brevis muscle. Axial T1 fat-saturated image post-gadolinium **B** showing homogeneous enhancement (blue arrow). Sagittal proton-density fat-saturated image **C** demonstrating an ovoid, high-signal, fusiform mass (green arrow) with a tapered fascicular continuity (orange arrow).

**Fig. 18:**
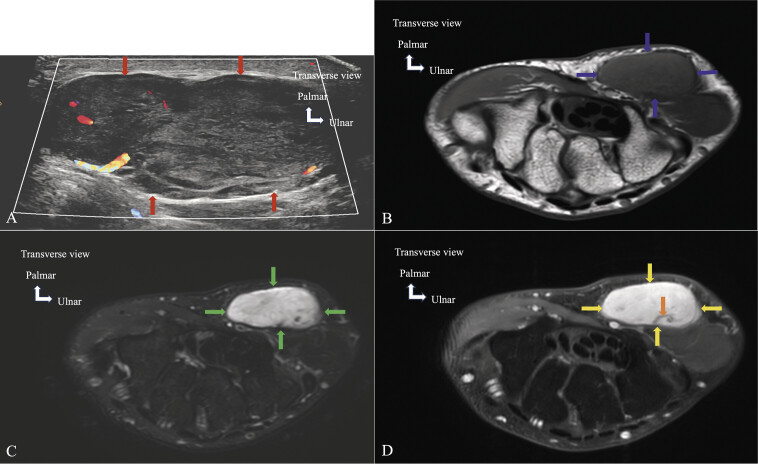
Angioleiomyoma. Transverse ultrasound image **A** demonstrating a heterogeneous and well-defined ovoid hypothenar mass (red arrows) with internal vessels. Axial T1-weighted image **B** demonstrating a well-defined ovoid mass (blue arrows). **C** Axial T2 Dixon water image demonstrating a heterogeneous mass with ovoid high T2 signal areas (green arrows). **D** Axial T1 fat-saturated image post-gadolinium demonstrating enhancement (yellow arrows) with flow void (orange arrow).

**Fig. 19:**
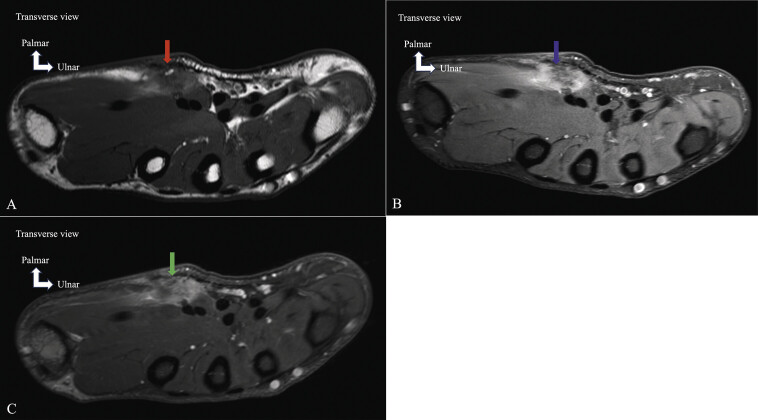
Epithelioid sarcoma of the thenar eminence. Axial T1-weighted image **A** demonstrating a heterogeneous thenar mass infiltrating the palmar aponeurosis and subcutaneous tissue (red arrow). Axial proton-density fat-saturated image **B** demonstrating a heterogeneous thenar mass with périlesional edema (blue arrow). Axial T1 fat-saturated image post-gadolinium **C** showing homogeneous enhancement and infiltrating the adjacent soft parts (green arrow).

X-rays are a good screening technique and can demonstrate radiopaque foreign bodies, such as glass, metal, and bone. US shows a hyperechoic structure with a posterior acoustic shadow. Thorns or splinters have a linear appearance. On Doppler, vascularization is often intense. Abscesses appear as heterogeneous fluid collections with ill-defined contours and peripheral hypervascularity 56 . On MRI, a foreign body granuloma appears as a poorly defined mass with a high T2 signal and an intermediate/low T1 signal. The foreign body may appear as a T2 hypointense signal. A ring-like peripheral enhancement of granulation tissue is possible after gadolinium injection 56 57 . Tenosynovial giant cell tumors are the most common solid tumors occurring in the hand. They are divided into localized and diffuse types 58 . Patients typically present between the ages of 20 and 50 years with a firm, immobile mass, with women being more affected 56 . In the hand, up to 85% of cases occur in the fingers, with the flexor tendon sheaths being involved more often than the extensor sheaths 56 . Localized tenosynovial giant cell tumors are the most common form in the hand. The diffuse form is less common. Tenosynovial giant cell tumors can be locally aggressive and can transform into a malignant tumor with metastasis. X-rays look for erosions or periosteal reactions. On US, a localized tenosynovial giant cell tumor appears as a hypoechoic mass with a homogeneous or heterogeneous texture 59 . It is attached to the tendon sheath but does not usually move with the tendon during dynamic evaluation. The contours are either sharp or confused with adjacent tissues. There is often intralesional Doppler flow 56 . On MRI ( Fig. 15 ), a localized tenosynovial giant cell tumor is usually heterogeneous, with a T1-weighted hypointense signal and an intermediate to low T2-weighted signal, indicating the presence of hemosiderin 58 . It may present with a blooming artifact on gradient echo sequences secondary to hemorrhage. The other most common tumors are lipomatous tumors ( Fig. 16 ), fibromatoses, neural tumors ( Fig. 17 ), fibromas of the tendon sheath, vascular tumors ( Fig. 18 ), and glomus tumors 56 . Soft-tissue sarcomas of the hand are rare. The most common subtypes are liposarcoma, synovial sarcoma, conventional epithelioid sarcoma ( Fig. 19 ), rhabdomyosarcoma, and clear cell sarcoma 56 . It is difficult to differentiate between the different subtypes because they can share clinical, imaging, and histologic features. Synovial sarcomas are usually encountered in the third to fourth decade of life. They tend to occur near tendons and joints and are not intra-articular. They are usually a slow-growing mass 56 . Small lesions may be confused with a benign mass because they are homogeneous and have well-defined boundaries 60 . On MRI T1-weighted images, they are usually isointense with respect to skeletal muscle and may have a heterogeneous T2 signal secondary to hemorrhage 61 . Fluid-fluid levels are possible, and there may be infiltration of adjacent tissues and bone.

Diagnosis of tumors and tumor-like lesions mainly relies on observational studies, case series, and imaging analyses. Ultrasound and MRI are essential for characterizing these masses, whether benign or malignant 51 52 53 54 55 56 57 58 59 60 61 . Due to the rarity and diversity of lesions, the available data are mostly descriptive and based on clinical experience.

Table 1 summarizes the main pathologies of the IMH discussed in this review, highlighting the key ultrasound and MRI findings for each, the most appropriate imaging sequences or techniques, and common diagnostic pitfalls.

**Table:** **Table 1** Pathologies affecting the intrinsic muscles of the hand – key imaging features, techniques, and diagnostic pitfalls.

Pathology	Key US/MRI findings	Most useful technique/MRI sequences	Common pitfalls
Muscle Denervation	MRI: homogeneous STIR hyperintensity in the acute phase; atrophy and fatty degeneration in the chronic phase. US: early hypoechoic appearance; late hyperechoic appearance	MRI: STIR and T1 sequences for staging assessment	STIR hyperintensity may be mistaken for an inflammatory or tumoral lesion Anatomical variants with dual innervation
Myositis	MRI: diffuse T2/STIR hyperintensity (often multicompartmental), enhancement after contrast injection (T1 fat-sat) In case of abscess: T2 hyperintensity, T1 hypointensity, peripheral enhancement	MRI T2/STIR for edema Contrast-enhanced T1 fat-sat to differentiate inflammation from abscess	Possible confusion with denervation edema MRI appearance often nonspecific without clinical context
Hand Compartment Syndrome (acute or exertional)	Key signs on imaging: Diffuse muscle edema, intramuscular hemorrhage	MRI T2/STIR for edema, gradient US for hemorrhage	Nonspecific clinical signs Anatomical variability of the compartments Risk of delayed diagnosis due to lack of consensus Compartment pressure measurement (Delta P < 30 mmHg) is controversial
Intrinsic Muscle Trauma	MRI: Non-systematized muscle edema, tendon avulsions (e.g., abductor pollicis brevis, interosseous muscles) US visualizes distraction injuries, differentiates muscular strain from tendon avulsion, and assesses associated lesions	MRI T2/STIR: edema Dynamic US: detailed evaluation of ears/distractions and adjacent structures	Confusion with denervation edema (non-systematized distribution) Rare and often unrecognized lesions (e.g., VIOM 3)
Saddle Syndrome (Syndrome interosseous-lumbrical)	Localized edema at the interosseous-lumbrical junction Scar tissue/adhesions visible on MRI	MRI T2/STIR: detection of edema and adhesions No specific US findings	Diagnosis often overlooked or missed Can be confused with isolated intrinsic muscle tension
Specific Lumbrical Muscle Pathologies	Edema or injury of the 3rd and 4th lumbrical muscles Lumbrical muscle enhancement in rheumatoid arthritis Possible selective denervation Abnormalities related to the “quadriga effect” in climbing	MRI T2/STIR: detection of edema and muscle lesions Contrast-enhanced T1 MRI: for lumbrical muscle enhancement in rheumatoid arthritis	Rare lesions, often underdiagnosed Possible confusion between traumatic lesions and neuropathies
Specific Interosseous Muscle Pathologies	Tenosynovitis of interosseous tendons (hand) in rheumatoid arthritis Often associated with metacarpophalangeal joint synovitis, sometimes isolated Tendon involvement in Dupuytren’s disease	Doppler US: detection of tenosynovitis MRI T2/STIR: tendon inflammation	Possible confusion with other causes of tendon or synovial inflammation
Tumors and Tumor-like Lesions	Cysts: anechoic/hypoechoic mass with thin walls, no Doppler vascularization Foreign body granulomas: poorly defined mass, high T2 signal, intermediate/low T1 signal; sometimes T2 hypointense signal for the foreign body itself Tenosynovial giant cell tumor: hypoechoic mass with intralesional Doppler flow; on MRI, hypointense on T1 and variable T2 signal due to hemosiderin Others: lipomas, fibromatoses, nerve tumors, sarcomas	US with Doppler MRI: T1, T2, fat-suppressed sequences, gradient echo sequences, contrast-enhanced T1 fat-sat	Confusion between benign and malignant lesions Small homogeneous lesions may be mistaken for benign masses Foreign bodies sometimes difficult to locate without a history of trauma
MRI: Magnetic Resonance Imaging; US: Ultrasound; STIR: Short Tau Inversion Recovery; VIOM: Volar Interosseous Muscles.			

This review study has certain limitations. It is a single-center study based on our personal experience and the institutional workflow for the management of IMH pathologies.

Some pathologies, such as traumatic tendon injuries and distraction injuries, are extremely rare and require studies that include multiple cases to better characterize them.

Finally, US is rarely used for the study of these IMH, and we believe that thorough knowledge of the anatomy and anatomical variants is necessary to perform it routinely and to benefit from its unique dynamic contribution.

## Conclusion

This review article focuses on the imaging of IMH pathologies. Good knowledge and understanding of the anatomy, anatomical variants, and pathologies specific to these muscles, including muscle denervation in MN or UN tunnel syndromes, myositis, rheumatic diseases, trauma, or tumor or pseudotumoral masses, facilitates high-resolution US and high-field T3 MRI assessments. In this regard, the improvement in US and MRI resolution is promising for the assessment of IMH. We hope that our contribution to understanding IMH pathologies will help clinicians better recognize and address pathological features. Finally, this review could serve as a guide for radiologists and surgeons for more advanced research studies.
